# Exploring the Chemical Space of CYP17A1 Inhibitors Using Cheminformatics and Machine Learning

**DOI:** 10.3390/molecules28041679

**Published:** 2023-02-09

**Authors:** Tianshi Yu, Tianyang Huang, Leiye Yu, Chanin Nantasenamat, Nuttapat Anuwongcharoen, Theeraphon Piacham, Ruobing Ren, Ying-Chih Chiang

**Affiliations:** 1 Kobilka Institute of Innovative Drug Discovery, School of Medicine, The Chinese University of Hong Kong (Shenzhen), Shenzhen 518172, China; 2 Center of Data Mining and Biomedical informatics, Faculty of Medical Technology, Mahidol University, Bangkok 10700, Thailand; 3 Shanghai Key Laboratory of Metabolic Remodeling and Health, Institute of Metabolism and Integrative Biology, Fudan University, Shanghai 200438, China; 4 Streamlit Open Source, Snowflake Inc., San Mateo, CA 94402, USA; 5 Department of Clinical Microbiology and Applied Technology, Faculty of Medical Technology, Mahidol University, Bangkok 10700, Thailand; 6 Shanghai Qi Zhi Institute, Shanghai 200030, China

**Keywords:** CYP17A1, prostate cancer, cheminformatics, quantitative structure–activity relationship, Murcko scaffold

## Abstract

Cytochrome P450 17A1 (CYP17A1) is one of the key enzymes in steroidogenesis that produces dehydroepiandrosterone (DHEA) from cholesterol. Abnormal DHEA production may lead to the progression of severe diseases, such as prostatic and breast cancers. Thus, CYP17A1 is a druggable target for anti-cancer molecule development. In this study, cheminformatic analyses and quantitative structure–activity relationship (QSAR) modeling were applied on a set of 962 CYP17A1 inhibitors (i.e., consisting of 279 steroidal and 683 nonsteroidal inhibitors) compiled from the ChEMBL database. For steroidal inhibitors, a QSAR classification model built using the PubChem fingerprint along with the extra trees algorithm achieved the best performance, reflected by the accuracy values of 0.933, 0.818, and 0.833 for the training, cross-validation, and test sets, respectively. For nonsteroidal inhibitors, a systematic cheminformatic analysis was applied for exploring the chemical space, Murcko scaffolds, and structure–activity relationships (SARs) for visualizing distributions, patterns, and representative scaffolds for drug discoveries. Furthermore, seven total QSAR classification models were established based on the nonsteroidal scaffolds, and two activity cliff (AC) generators were identified. The best performing model out of these seven was model VIII, which is built upon the PubChem fingerprint along with the random forest algorithm. It achieved a robust accuracy across the training set, the cross-validation set, and the test set, i.e., 0.96, 0.92, and 0.913, respectively. It is anticipated that the results presented herein would be instrumental for further CYP17A1 inhibitor drug discovery efforts.

## 1. Introduction

Prostate cancer is highly prevalent in men worldwide. The androgen–androgen receptor axis is essential for disease progression. Thus, androgen-deprivation therapy, either medically or surgically, is a front-line prostate cancer treatment. However, after a median of 24 months of androgen-deprivation therapy, treatment resistance is inevitable, as demonstrated by a relapse in serum prostate-specific antigen levels [1]. Dehydroepiandrosterone (DHEA), secreted from the adrenal gland, fuels the development of castration-resistant prostate cancer (CRPC). Cytochrome P450 17A1 (CYP17A1) is essential for the generation of DHEA from cholesterol. Abiraterone acetate, termed abiraterone hereafter, was approved for CRPC treatments in 2011, highlighting the importance of CYP17A1 in prostate cancer management.

A series of small molecules has been reported as CYP17A1 inhibitors. Ketoconazole is the first nonsteroidal CYP17A1 inhibitor used for prostate cancer treatment, with poor selectivity and mild androgen receptor agonist effects [2]. Abiraterone, with a similar steroidal scaffold to the natural substrates of CYP17A1 (pregnenolone or progesterone), inhibits both the 17,20-lyase and 17α-hydroxylase activities of CYP17A1 more potently. However, the inhibition of 17α-hydroxylase activities prevents the generation of cortisol, leading to side effects, including hypertension and fluid overload [3]. Prednisone is co-administered with abiraterone to supplement cortisol defects. Seviteronel and orteronel have been developed as the selective nonsteroidal inhibitors of 17,20-lyase [2]. In preclinical studies, both drugs preferentially inhibited 17,20-lyase over 17α-hydroxylase, thus offering a potential advantage over abiraterone from the perspective of not requiring concomitant therapy with prednisone. Galeterone is a steroidal CYP17A1-lyase inhibitor with modifications on the D-ring at the C17 position of abiraterone [4]. Galeterone has also been reported to have multiple mechanisms of action, including CYP17A1 inhibition, androgen receptor antagonism, and a decrease in intratumoral androgen receptor levels. In clinical trials, orteronel and galeterone showed promising but insufficient effects in prostate cancer patients (SWOG-1216; ARMOR 1 and 2), indicating that further drug optimizations should be performed. 

Computer-aided drug discoveries are indispensable operations in the entire process of drug discovery. As a multidisciplinary field, it involves data science, statistics, pharmacology, medicinal chemistry, structural biology and computational biology, etc. Ligand-based drug discoveries are common approaches used in computer-aided drug discovery. This focuses on ligands, which are chemical entities that can bind to the biological target, while information with respect to the target is not required. Ligand-based drug discovery can be applied to identify hits or to optimize hits/leads using two important approaches, namely the quantitative structure–activity/property relationship (QSAR/QSPR) and pharmacophore modeling [5]. The former is a mathematical model used for correlating the bioactivities or physicochemical properties of molecules with their (sub)structures. The latter attempts to identify the interactions between a ligand and its receptor.

The essence of QSAR is based on two major principles: (i) structure dictates activity and (ii) molecules with similar structures demonstrate similar bioactivities or properties. As a methodology in chemistry and drug discovery, QSAR/QSPR experienced remarkable transformations since its introduction 60 years ago. As early as the time of Corwin Hansch, QSAR/QSPR modeling operations were performed on a small number of molecules, with few molecular descriptors, employing multilinear regression. Now, thanks to the development of information technology and artificial intelligence, QSAR/QSPR has evolved with respect to the application of a large dataset, equipped with sophisticated molecular descriptors, advanced machine learning algorithms, and various validation techniques. The Organization of Economic Cooperation and Development established principles for QSAR modeling consisting of five rules: defined endpoint, unambiguous algorithms, defined applicability domain, modeling validation, and mechanistic interpretation, if possible. This involves various steps, including data compilation, data splitting, the machine learning process, an evaluation of the robustness and predictability of the model, and the mechanistic interpretation of feature importance [6,7,8], etc.

In this study, we performed systematic cheminformatic analyses and machine learning modeling techniques to explore the chemical space of CYP17A1 inhibitors. By separating the activities into four categories, viz., potent (pIC_50_ ≥ 8), active (8 > pIC_50_ ≥ 7), intermediate (7 > pIC_50_ ≥ 6), and inactive (pIC_50_ < 6), we built one QSAR classification model (model I) to reveal the interrelation between steroidal inhibitor structures and the associated activities. The structures were represented using PubChem and KlekotaRothCount fingerprints provided by the PaDEL package [9].

For nonsteroidal inhibitors, we visualized the associated chemical space, extracted the representative scaffolds, analyzed their diversity, and constructed seven classification QSAR models (model II to model VIII, according to the scaffolds) for bioactivity predictions. The findings in this study can provide guidance, leading the optimization of further CYP17A1 inhibitor drug discoveries.

## 2. Results

### 2.1. Exploratory Data Analysis Visualization 

**Figure 1:**
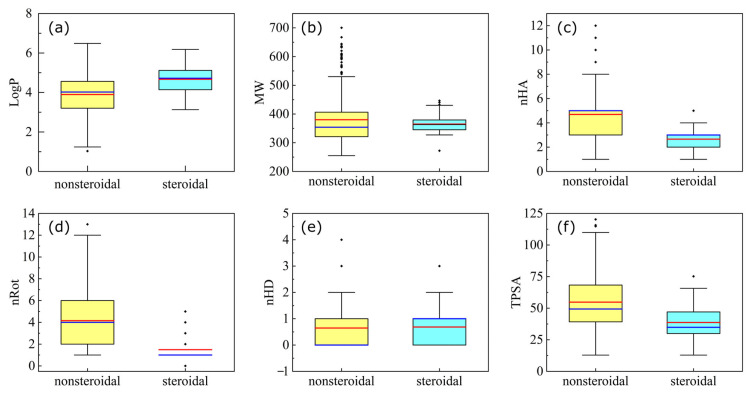
Box plot of drug likeness descriptors amongst nonsteroidal and steroidal inhibitors. Only potent/active molecules in nonsteroidal and steroidal categories were selected for the comparison. The average and the median are indicated by the red and blue lines, while the lower and upper quartiles are labeled by the lower and upper boundaries of the box, respectively. Panel (**a**–**f**) show the distributions of LogP, MW, nHA, nRot, nHD, and TPSA, respectively.

Exploratory data analysis was performed to compare nonsteroidal and steroidal molecules based on six drug-likeness descriptors, including the molecular weight (MW), the octanol–water partition coefficient (LogP), the number of hydrogen bond acceptors (nHA), the number of hydrogen bond donors (nHD), the number of rotatable bonds (nRot), and the topological polar surface area (TPSA). Only potent and active inhibitors (pIC_50_ ≥ 7.0) were employed in the analysis. Results are shown in Figure 1 and Table 1. All properties demonstrated nonparametric distribution patterns. Significant differences between nonsteroidal and steroidal inhibitors were identified in properties, such as LogP, nHA, nRot, and TPSA, while the U test showed no or less significant differences between the nonsteroidal and steroidal inhibitors in the properties of MW and of nHD. Table 1 shows these details. Nonsteroidal inhibitors often had a smaller LogP but larger nHA, nRot, and TPSA in comparison with those of the steroidal ones, as shown in Figure 1. Notably, the nonsteroidal distributions on MW, nHA, nHD, and TPSA were also more skewed (less symmetric), as reflected by their skewness. All distributions were platykurtic distributions (diversely distributed) since all kurtosis measures were smaller than 3. Nevertheless, the nonsteroidal distributions of nHA and nHD still showed much larger kurtoses than the steroidal ones.

### 2.2. Chemical Space Visualization via Principal Component Analysis

**Figure 2:**
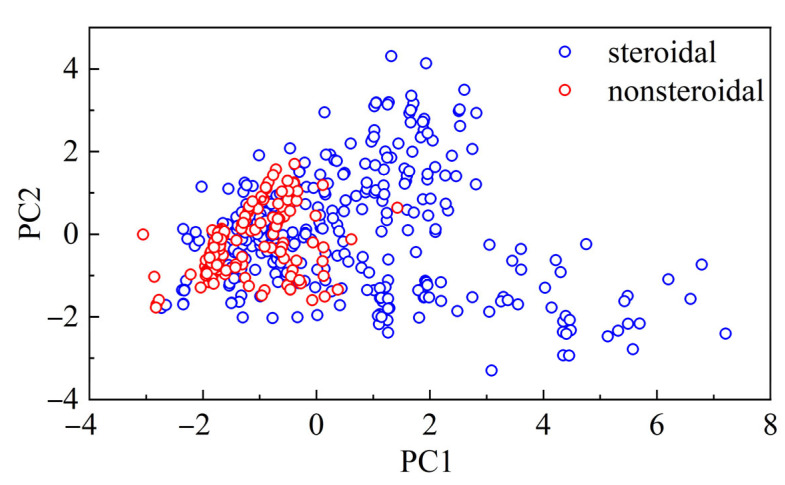
Chemical space occupied by nonsteroidal molecules (blue circle) and steroidal molecules (red circle) and spanned by the first two principal components (PC1 and PC2). The analysis was performed for all molecules regardless of their bioactivity classes. The detailed compositions of PC1 and PC2 are provided in Table 2. In comparison with nonsteroidal data, steroidal data were centered in a much smaller area.

Principal component analyses (PCAs) were performed over the distribution of the six drug-likeness properties, as mentioned in Table 1, with all molecules. By projecting the data onto the first two principal components (PC1 and PC2), one can visualize the chemical space occupied by the nonsteroidal molecules and by the steroidal molecules. As depicted in Figure 2, nonsteroidal data distributions were significantly different from steroidal data. The former spread widely and overlapped with the latter, which only occupied a small area in the plot. This implies that the chemical structure, or the scaffolds of molecules, are more diverse for the nonsteroidal compounds than for the steroidal compounds. Such visualization is valid because the top two components (PC1 and PC2) already accounted for 82.6% of the variance. Introducing the third principal component (PC3) will increase the accumulated variance to about 94.3%. The detailed component compositions and the accumulated variance are listed in Table 2. The first component PC1 mostly comprised nHA (0.535), TPSA (0.516), and nRot (0.494), followed by MW (0.383) and LogP (−0.218). In contrast, PC2 mostly comprised nHD (0.578), LogP (−0.597), and MW (−0.486), followed by TPSA (0.210) and nRot (−0.159). Notably, PC1 and PC2 had very low contributions from nHD and nHA, respectively, while PC3 had a dominant contribution from nHD (0.757).

### 2.3. QSAR Modeling and Validation for Steroidal Inhibitors

After the initial analysis, a classification QSAR model (model I) was built to predict the bioactivity class for a steroidal compound. PubChem fingerprints were employed for generating the fingerprint information of steroidal CYP17A1 inhibitors, and 12 representative classification algorithms were tested to construct the model with the best performance. The tested algorithms included the decision tree (DT), extra trees (ET), random forest (RF), gradient boost (GB), light gradient boosting machine (LGBM), extreme gradient boost (XGB), multilayer perceptron (MLP), logistic regression (LR), K-nearest neighbor (KNN), support vector machine (SVM), naïve-Bayes (NB), and Gaussian process (GP) algorithms. The descriptions for these algorithms are provided in the Materials and Methods (Section 4.5.3). The performances of the algorithms were evaluated via the accuracy and Matthew’s correlation coefficient (MCC). These two quantities are defined using the true positive (TP), true negative (TN), false negative (FN), and false positive (FP). 

**Figure 3:**
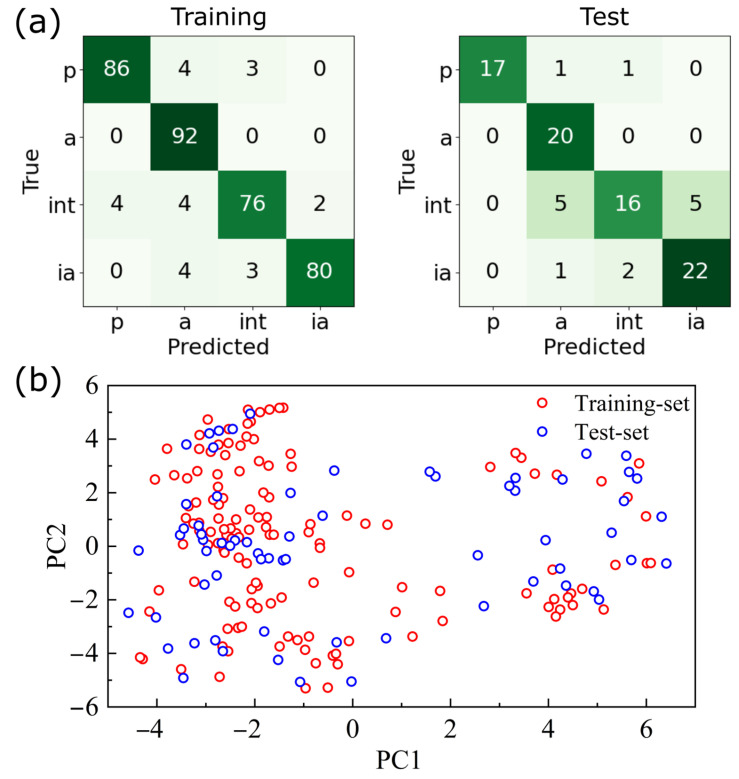
(**a**) Confusion matrices of model I for the training set and the test set. Labels p, a, int, and ia stand for potent, active, intermediate, and inactive compounds, respectively. The prediction was quite good, as the diagonal values are much larger than the off-diagonal ones. (**b**) Visualization of the applicability domain of model I. The distributions of the training set (red circle) and of the test set (blue circle) in the chemical space are depicted, spanned based on principal components PC1 and PC2. As the two sets of data are distributed over the same area in the chemical space, the QSAR model constructed with the training set should predict the bioactivity well for the test set, i.e., the test set falls within the applicability domain.

The performances of the 12 algorithms are listed in Table 3. The results are shown separately for the training set (labeled as Training), the 10-fold cross-validation set (labeled as CV), and the test set (labeled as Test). According to this table, ET provides the best performance with an accuracy of 0.933, 0.818, and 0.833 for the training data, the 10-fold cross-validation set, and the test set, respectively. This was selected as our QSAR model for the steroidal compounds (model I). The confusion matrices for the model I training set and test set are depicted in Figure 3a. The diagonal elements are the TP for each class. The sum of each row gives the TP+FN for each class, while the sum of each column yields the TP+FP for each class. Finally, the sum over the matrix without a class’s column and row gives the TN for that class. Based on these observations, one can prove that the multiclass accuracy reported in Table 3 is the same as the multiclass micro-averaged recall; see Section 4.5.4 for more details. Thus, there is no need to report the micro-averaged recall separately.

Notably, algorithms, such as RF, DT, XGB, LGBM, GB, MLP, and GP, also performed quite well. Now, one may notice that the performance of the test set is quite good and is comparable with the performance of the cross-validation set or even the performance of the training set. This can be explained by how these data are distributed in the chemical space. Figure 3b shows the distributions of the training data and test data in the chemical space, spanned based on principal components PC1 and PC2. The distribution of the test data overlapped nicely with the distribution of the training data, demonstrating that the test set falls within the applicability domain. In other words, successful bioactivity predictions can be achieved, if the QSAR model is constructed and applied only for molecules with the same scaffold.

### 2.4. Murcko Scaffold Analysis and R-Group Decomposition-Based Structure–Activity Relationship for Nonsteroidal Inhibitors

Following the previous success in QSAR modeling for steroidal compounds, we planned to carry out the same modeling operation for nonsteroidal molecules. However, the chemical structures of nonsteroidal molecules are more diverse, i.e., there are many different scaffolds. Thus, the underlying scaffolds must be identified before the QSAR models can be constructed. To achieve this, we visualized the nonsteroidal scaffolds, analyzed the level of diversity, and calculated the scaffold enrichment factor.

Table 4 provides the overview of different types of scaffolds found in the nonsteroidal inhibitors. Generally, molecules with a pIC_50_ ≥ 7.0 (active and potent molecules) demonstrated lower scaffold diversity than those with a pIC_50_ < 7.0. Hence, finding more novel scaffolds for androgen receptor antagonists is desired.

**Figure 4:**
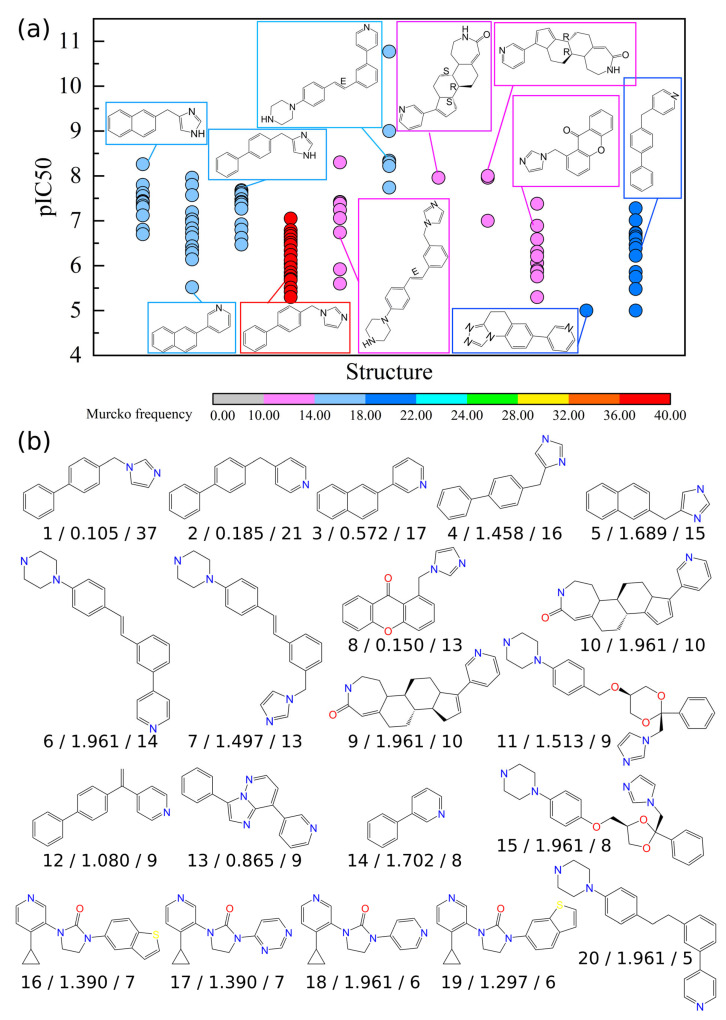

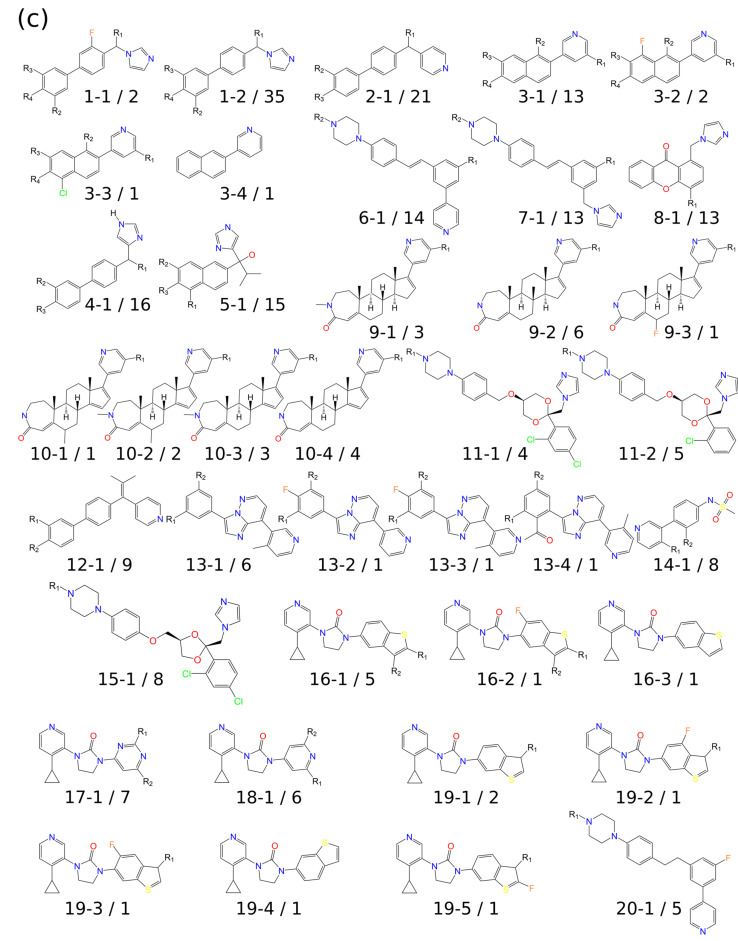
Scaffold analysis of nonsteroidal inhibitors. (**a**) Murcko scaffold vs. pIC_50_ as a scatter plot for 683 nonsteroidal inhibitors. Only Murcko frequencies that are ≥10 are demonstrated, and the complete scatter plot is shown in Figure S1 from the Supplementary Materials. (**b**) Representative scaffolds with either a frequency ≥ 10 or EF ≥ 1. Scaffolds are labeled as XX/YY/ZZ, where XX, YY, and ZZ denote the scaffold’s ID, EF, and the frequency of occurrence, respectively. (**c**) Detailed list of Murcko-based core fragments from representative Murcko scaffolds. Fragments are labeled with the scaffold ID, core fragment ID, and the occurrence frequency (scaffold ID—core fragment ID/frequency).

Using Murcko scaffolds, nonsteroidal molecules can be sorted based on their structures. The distribution of the bioactivity (pIC_50_) thus can be visualized for each scaffold. Figure 4a shows the pIC_50_ of scaffolds with a frequency of occurrence (Murcko frequency) equal to or larger than 10. Among the 11 scaffolds in the figure, 10 of them showed datapoints with a pIC_50_ ≥ 7, viz., a scaffold containing active or potent compounds. In particular, some scaffolds contained only active or potent molecules, e.g., structures in the middle of the panel with all datapoints with a pIC_50_ ≥ 7. These scaffolds are worthy of further analyses. A complete scatter plot of the Murcko scaffold vs. pIC_50_ for 683 nonsteroidal inhibitors can be found in the SI (Figure S1). On the other hand, scaffolds with Murcko frequencies that are <10 could still be hidden gems for new drug development if they have a good scaffold enrichment factor (EF). The EF is the ratio of the proportion of active molecules with a given scaffold to the proportion of active molecules in the entire dataset. Thus, EF ≥ 1 means that there are more active/potent molecules in the ratio used in the current scaffold than that used on average. Hence, based on these two criteria, 20 Murcko scaffolds were selected for further analysis. Their structures are depicted in Figure 4b. Among the 20 scaffolds, scaffold ID 6, 9, 10, 15, 18, and 20 had the highest EF value 1.961. These are scaffolds with all molecules being active or potent against CYP17A1. These are the most favorable scaffolds. On the other hand, scaffolds 4, 5, 7, 11, 12, 14, 16, and 17 had an EF between 1 and 1.961, indicating that they are also favorable scaffolds but just not as favorable as the previous ones. Finally, scaffolds 1, 2, 3, 8, and 13 had an EF smaller than 1, meaning that they are unfavorable scaffolds with a proportion of potent/active molecules less than the one from the entire dataset. 

Based on the 20 scaffolds, we can further discuss how the functional group substitution affects the bioactivities. For this purpose, the core fragments of each scaffold are depicted in Figure 4c. These Murcko-based core fragments were obtained via the automatic structure–activity relationship (SAR) analysis from DataWarrior, and they are labeled by the scaffold ID and core ID, e.g., X-Y represents the core fragment Y from scaffold X. The detailed impact of functional group substitutions on bioactivities is listed in Table 5. Here, we briefly mention some special cases with highly favorable fragments:

1. Scaffold 1 has two core fragments. Fragment 1-1 has fluoride and all molecules within this group are active. Fragment 1-2 generally has weaker bioactivities than fragment 1-1, and its functional group substitution is provided in Table 5.
2. Scaffold 6 only has one core fragment (6-1). All molecules in this scaffold series are strong inhibitors regardless of the substitutions.
3. Scaffolds 9 and 10. These scaffolds are steroid-like, with ring A replaced by a seven-membered ring. All molecules in these scaffolds are highly active against CYP17A1 regardless of the substitutions.
4. Scaffold 14 only has one core fragment (14-1). Molecules with methyl groups on the R1 or R2 positions are active.
5. Scaffold 15 only has one core fragment (15-1). All molecules in this scaffold are potent or active against CYP17A1. All functional groups at the R1 position are sulfonyl groups.
6. Scaffolds 16, 17, 18, and 19 are similar scaffolds sharing the same cyclic skeleton. All molecules in scaffold 18 are either potent or active against CYP17A1 regardless of the substitution. For other scaffolds, compounds with fluoride-containing groups on R1 or R2 positions are active.
7. Scaffold 20 has only one core fragment (20-1). All molecules in this scaffold are potent or active against CYP17A1, and all functional groups on the R1 position contain the carbonyl group.

### 2.5. QSAR Models of Nonsteroidal Inhibitors Based on Murcko Scaffold Analysis

**Figure 5:**
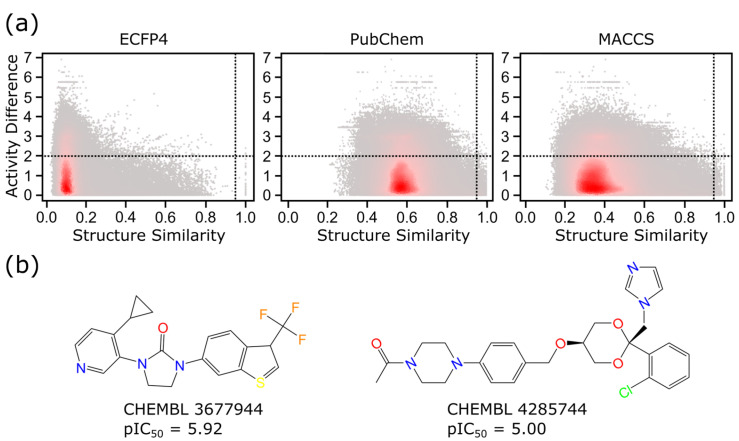
Structure-activity landscape of nonsteroidal inhibitors. (**a**) SAS maps of nonsteroidal molecules with three different fingerprints. Regions with densely populated molecular pairs are colored in red, while less populated regions are colored in gray. The dashed lines outline the boundaries for the activity difference and the structure similarity. Any pairs appearing in the right upper quadrant were identified as ACs. (**b**) Maximum common AC generators from the PubChem and MACCS fingerprints.

Prior to QSAR modeling for nonsteroidal inhibitors, structure–activity similarity (SAS) maps were visualized to identify activity cliffs (ACs); i.e., molecules have highly similar structures but show large activity differences. ACs are cases where the SAR is violated and thus should be avoided in QSAR modeling processes. As shown by the SAS maps of nonsteroidal compounds, viz., Figure 5a, most densely populated molecular pairs (red region) appeared to have lower structural similarities. However, discontinuities between the structure’s similarity and the activity were spotted by using three sets of fingerprints. Extended-connectivity fingerprint 4 (ECFP4) had the lowest density in the AC quadrant (the right upper quadrant formed by dash lines), while the molecular access system (MACCS) and PubChem fingerprints had an increased density. Luckily, the AC quadrants occupied only very small and marginal regions of SAS maps; thus, we could proceed to build the QSAR models. For the sake of completeness, the maximum common AC generators between PubChem and MACCS fingerprints are shown in Figure 5b. AC generators are molecules that appear frequently in the activity cliff regions of the SAS map. ECFP4 did not possess any AC generators. The examples of the AC pairs are listed in SI (Table S1).

**Figure 6:**
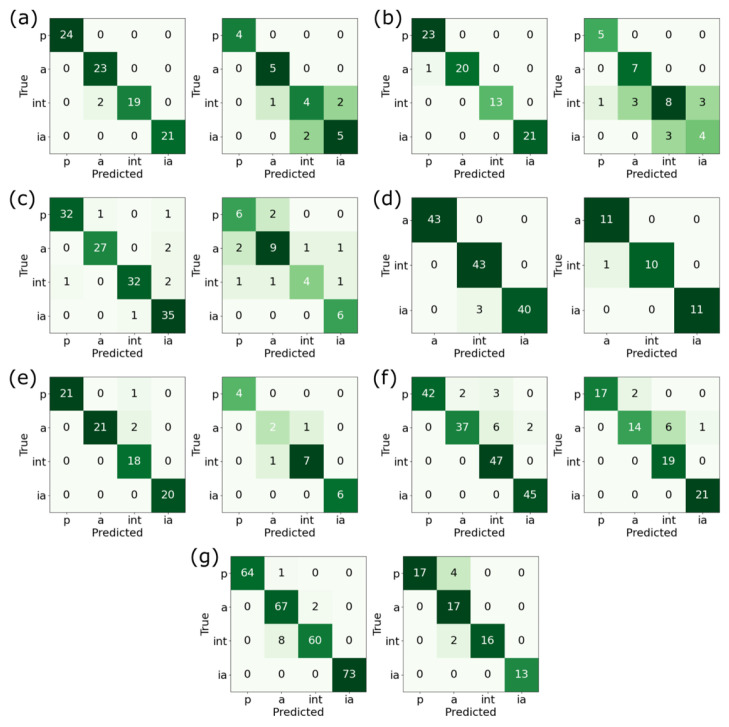
Confusion matrices for the QSAR models of nonsteroidal compounds. (**a**–**g**) Show the confusion matrices for the training set (left) and for the test set (right) of model II to model VIII, respectively. In comparison with the steroidal compounds (model I), nonsteroidal compounds were fewer in number. While the training performance was quite good overall, the test set confusion matrices in model II, III, and IV had quite large off-diagonal elements, compared with the associated diagonal values. This is also reflected by the accuracy reported in Table 6. Note that model V has no potent compounds. The associated matrices in panel (**d**) are therefore 3-by-3.

Now, we could construct the QSAR models for the nonsteroidal compounds. Previously, we identified 20 important scaffolds, but only 683 nonsteroidal molecular data were available for training. Hence, combining data from similar scaffolds, judged by their cyclic skeletons, for QSAR modeling is necessary. This yielded seven groups of cyclic skeletons: scaffolds 1 and 4; scaffolds 2 and 12; scaffolds 6, 7, 11, and 20; scaffold 3; scaffold 5; scaffolds 16, 17, 18, and 19; and scaffold 13. Consequently, seven QSAR models (model II to model VIII) were established for the seven groups. Again, 12 algorithms and 3 molecular fingerprints were tested and the combination with the best performance was selected as the QSAR model. The performance of the algorithms with the best choice of the fingerprint can be found in the SI (Table S2 to Table S8 for model II to model VIII, respectively). The best performing results, the accuracy of QSAR model II to VIII, are listed in Table 6, while the associated confusion matrices are depicted in Figure 6. Except for model VIII, all nonsteroidal models had significantly less data available than those for the steroidal compounds (model I). Nonetheless, QSAR models were still built, and acceptable performance for the test set was achieved: robust and reliable results were found in at least 5 out of 12 algorithms for each model. Notably, model V contained no potent compounds, see Figure 6d, hence it is not a good choice for developing a highly potent inhibitor. While these QSAR models are useful, there is still a preference. For example, models IV and VII contain scaffolds with the highest EF. The associated compounds can also be highly potent. These two QSAR models, hence, will be useful when pursuing high potency inhibitors against CYP17A1.

The reader may notice that no classification QSAR models were constructed for scaffolds 8, 9, 10, 14, and 15. Scaffolds 9 and 10 were steroidal analogs with EF = 1.961, meaning that all associated molecules are active or potent. Hence, there was no point in constructing the classification model, but they are still good candidates for drug development. Scaffold 8 was a structurally unique scaffold with three tandem-fused benzene rings. Only 16 molecules belonged to this scaffold. The count of molecules was too small to build robust models. The same problem occurred for scaffold 15. As for scaffold 14, its cyclic skeleton was too simple and can be found in 433 nonsteroidal molecules with highly diversified structures. After initial modeling with the molecules, the model demonstrated significant overfitting that cannot be used for predictions.

## 3. Discussion

CYP17A1 inhibitors have been structurally categorized as steroidal or nonsteroidal. The steroidal inhibitors are structurally similar to the natural substrates of CYP17A1, viz., pregnenolone or progesterone, and they are often involved in the modification of the substrate’s D-ring at the C17 position. A typical steroidal CYP17A1 inhibitor is abiraterone, which is currently administered in combination with prednisone for the treatment of metastatic CRPC in men. Unfortunately, as time proceeds, resistance to abiraterone is developed in most cases. Furthermore, abiraterone-resistant tumors are also frequently resistant to subsequent treatments with enzalutamide, an androgen receptor antagonist that otherwise could confer a survival benefit that is similar to that of abiraterone [2]. Galeterone is another steroidal CYP17A1 inhibitor. It has multiple mechanisms of action, including CYP17A1 inhibition, androgen receptor antagonism, and a decrease in intratumoral androgen receptor levels. Additionally, galeterone has a unique mechanism of action, mediated by disrupting AR signaling via a proteasome-dependent pathway, leading to androgen receptor degradation. In a phase I study of chemonaive men with CRPC, 22% demonstrated a decrease in prostate-specific antigens for more than 50%, whereas an additional 26% had a prostate-specific antigen decline of 30–50% after 12 weeks. No evidence for excess adrenal mineralocorticoid was noted. However, clinical trials for galeterone were discontinued because the expected survival rate was not met. Another drug with abiraterone-like properties is orteronel. It is a nonsteroidal selective inhibitor of 17,20-lyase. In preclinical studies, the inhibitory effect of orteronel on 17,20-lyase was 5.4-fold greater than that on 17α-hydroxylase, with minimal effects on other CYP drug-metabolizing enzymes. Preliminary results from the phase I/II clinical trials demonstrated a 63% of the prostate-specific antigen response rate at 12 weeks. The advantage of orteronel lies in its selectivity, which results in a reduction in the risk of the overproduction of mineralocorticoids. However, clinical trials were finally discontinued as orteronel failed to meet the expected overall survival [10]. The last drug discussed here is seviteronel, which is a novel nonsteroidal CYP17A1 inhibitor and androgen receptor antagonist. It preferentially inhibits 17,20-lyase over 17α-hydroxylase, but the clinical trials were terminated due to unsatisfactory tolerance and clinical responses [4,11,12]. It is important to note that both orteronel and seviteronel share the same scaffold, viz., scaffold 5 shown in Figure 4b. The SAR-based enumeration of more structural analogs can be synthesized to identify better alternatives. 

The most challenging issue of treating CRPC with abiraterone is its resistance. Currently, there are several hypotheses to explain the emerging resistance to abiraterone, including androgen receptor splice variant up-regulation of CYP17A1; the increased expression of steroidogenic enzymes, including AKR1C3 and HSD17B3 [13]; 17-hydroxyprogesterone being transformed into the 5α-dione pathway [14]; androgen receptor activation mediated by exogenous corticosteroids during the treatment; the interruption or reversal of DHT levels; the miRNA modulation of the androgen receptor pathway; and the involvement of PI3K/AKT/mTOR pathways [15]. The resistance to abiraterone, along with the side effects and sophisticated pathogenic mechanisms of CRPC, pose urgent needs for new drug discovery. Apart from resistance, the administration of abiraterone to patients can also trigger off-target effects due to the steroidal nature of abiraterone. It is important to note that the current efforts in drug discovery are devoted to nonsteroidal inhibitors to avoid off-target effects [16].

There are a few previous studies focusing on the structure–activity relationship of CYP17A1 inhibitors [17,18,19]. Previous studies focused on singular scaffolds or worked through 3D pharmacophore models. This study used comprehensive datasets from the ChEMBL database, with 279 steroidal molecules and 683 nonsteroidal molecules that cover dozens of scaffolds.

Additionally, this study identified several significant ACs and AC generators. AC generators are defined as molecules that frequently appear among AC molecule pairs that are identified by the structure–activity landscape index (SALI) values. The identification of AC generators amongst datasets are important for medicinal chemists to facilitate further lead optimizations. The two maximum common AC generators shown in Figure 5b are the common AC generators between the PubChem and MACCS fingerprints. ECFP4 does not possess AC generators. AC generators CHEMBL 3677944 and CHEMBL 4285744 belong to scaffold 19 and scaffold 11, respectively. Special attention should be focused on them when performing lead optimizations.

The applicability domain is a theoretical region in the chemical space surrounding both the model’s descriptors and modeled response. In the construction of a QSAR model, the applicability domain of molecules plays a critical role in estimating the uncertainty in the prediction of a given set of compounds based on how similar they are to the training sets used to build the model [8,20,21]. The prediction of molecular bioactivity using QSAR is applicable only if the molecule falls within the applicability domain of the model. In other words, the QSAR model built for a specific scaffold or skeleton should only be used to predict the bioactivities of molecules that pose the same scaffold/skeleton. It is unsuitable for predicting activities for all chemicals, using a single QSAR model. For this reason, all molecules used in this computational study were categorized based on steroidal and nonsteroidal compounds, followed by an in-depth analysis of nonsteroidal scaffolds. For nonsteroidal compounds, 20 representative scaffolds were extracted. These are scaffolds with a high frequency or high EF values. The QSAR models were then constructed based on the carefully chosen scaffolds or cyclic skeletons. In total, eight QSAR models were built: one for the steroidal compounds (model I) and seven for the nonsteroidal compounds. All models demonstrated robustness and reliability. While the QSAR models constructed here can be used to predict bioactivities for new chemical entities, the most valuable information revealed in this work is perhaps the knowledge learned from the scaffold analysis for nonsteroidal compounds. Not only were the highly potent nonsteroidal scaffolds identified, but the impact on functional group substitutions was also discovered. This provides valuable insights for further lead optimization or for the enumeration of new molecules. For example, nonsteroidal scaffolds 9, 10, and 15 are important scaffolds with only active or potent compounds reported. Although no QSAR model can be constructed for them, their scaffolds serve as good starting points for lead optimizations. Such information sometimes can be more valuable than a QSAR model. On the contrary, based on the EF values, scaffolds 1, 2, 3, 8, and 13 are less favorable for developing CYP17A1 inhibitors. Additionally, we noticed that several functional groups could affect the bioactivity upon substitution. They are the hydroxyl group, halogen, fluoride, sulfonamide, ketone, amide, and the methyl group. The effect, i.e., whether the group increases or decreases the inhibitory activity, depends on where and on which scaffold the substitution takes place. 

Finally, we mention that the current datasets are compiled from the ChEMBL database. These are retrospective records accumulated over years and with various sources for the bioactivities. Further prospective studies should focus on molecular enumerations based on scaffold analysis and SAR, molecular synthesis, and experimental validation. In addition, structural analysis based on ligand-protein interactions via molecular dynamic simulation could provide valuable insights into the mechanisms of action. 

## 4. Materials and Methods

**Figure 7:**
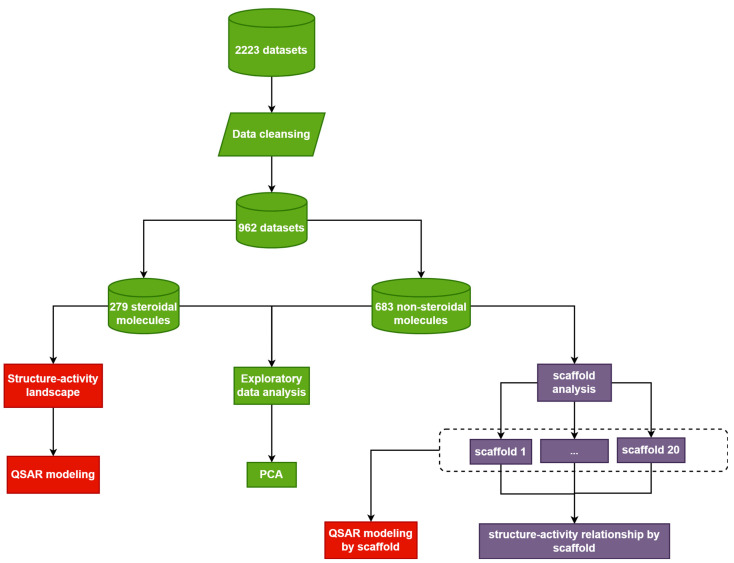
Schematic diagram of the study. Different colors represent different stages of the study: green for data compilation and visualization, red for structure–activity relationship/QSAR, and purple for scaffold analysis.

This was a computational study of CYP17A1 inhibitors. All steps and procedures were performed using computational methods. The study’s design consists of data compilation, exploratory data analysis, Murcko-scaffold analysis, structure–activity landscape visualization, and QSAR modeling, as shown in Figure 7.

### 4.1. Data Compilation

**Figure 8:**
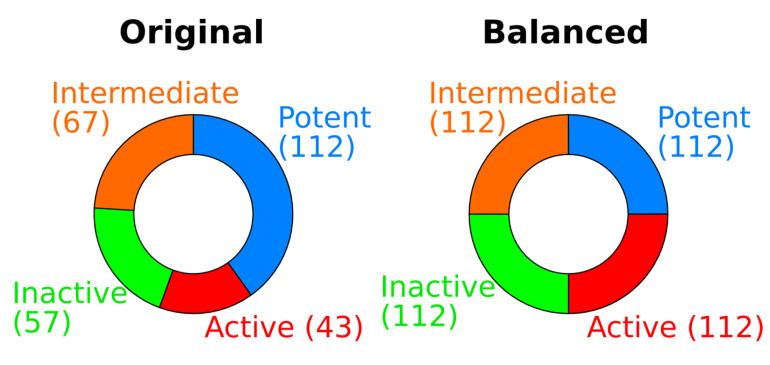
Frequency of bioactivity of 279 steroidal inhibitors. Using random oversampling strategies for data balancing, there were 448 datasets in total. The 448 datasets are the results of the random duplication of the three minor classes (active, intermediate, and inactive class) to match with major class (potent class).

The IC_50_ data used in this study were taken from the ChEMBL database. Originally, there were 2223 activity entries, but only 962 remained after the data cleansing procedure, which included removing redundant, unqualified, or missing data. The associated molecule structures were also processed to remove the salt and to standardize the tautomers using the PaDEL package [9]. Among the 962 molecules, 683 of them were nonsteroidal ligands, while the other 279 molecules were steroidal ligands. We further transformed the IC_50_ to pIC_50_ (−log IC_50_) for better visualization and data processing [22]. Molecules with pIC_50_ ≥ 8, 8 > pIC_50_ ≥ 7, 7 > pIC_50_ ≥ 6, and pIC_50_ < 6 were labeled as potent, active, intermediate, and inactive, respectively. As shown in Figure 8, the original datasets for steroidal inhibitors are highly imbalanced, e.g., the ratio of active ligands to potent ligands is 1: 2.6. To avoid any overfitting caused by imbalanced data, we further balanced the dataset via the oversampling technique. Specifically, we randomly selected and duplicated data within the active, intermediate, and inactive classes until their sizes equaled the size of potent data (112 entries). The same procedure applies to the seven other nonsteroidal QSAR models. 

### 4.2. Exploratory Data Analysis of Drug-Likeness Properties

In this study, exploratory data analyses of the datasets focused on the visualization of drug-likeness properties. Six drug-likeness properties were calculated, visualized, and compared between active class nonsteroidal inhibitors and active class steroidal inhibitors: MW, LogP, nHA, nHD, nRot, and TPSA. In this section, the maximal, minimal, median, mean, skewness, and kurtosis were analyzed for the descriptors, and normality tests were performed to observe whether they abide by normal distributions. Then, the *p*-values of the Mann–Whitney tests (if not normal distribution) between different groups were calculated to determine if there were any statistically significant differences. DataWarrior [23] was used for exploratory data analysis. 

### 4.3. PCA

The PCA is a dimensionality-reduction method that is used to reduce the dimensionality of large datasets by transforming large datasets into smaller datasets that still contain most of the information of the large set. In this study, the abovementioned 10 drug-likeness properties were used to perform PCA after exploratory data analysis had been performed. DataWarrior [23] was used for the PCA. 

### 4.4. Structure–Activity Landscape Visualization

SAR is based on the idea that structure dictates activity, and molecules with similar structures demonstrate similar bioactivities. However, this relationship can be interrupted by AC, where a small change in structure can result in substantial activity loss. Since ACs capture chemical modifications that strongly influence biological activity, they are of particular interest in drug discovery and must be applied prior to any SAR modelling. In this study, SAS maps and SALI [24] plots were used to visualize the structure–activity landscape and identify ACs. The SAS map is a pairwise 2D plot of the activity difference against structure similarity. The plot consists of 4 quadrants: smooth regions of the SAR space, rough region of activity cliffs, nondescript region (i.e., low structural similarity and low activity similarity), and scaffold hopping region (low structural similarity but high activity similarity). In this study, Activity Landscape Plotter V.1, a webserver to generate SAS maps, was used [25]. SALI provides the numerical quantification of ACs. The DataWarrior application was used to calculate the SALI value and generate the SALI plot of datasets.

### 4.5. QSAR Modeling

#### 4.5.1. Molecular Fingerprints

Molecular fingerprints are the representations of a complex form of molecular descriptors, which describe molecules in terms of their constitution, connectivity, and physicochemical properties. They are typically encoded by bit strings to characterize a given molecule. In this study, KlekotaRoth and PubChem fingerprints provided by the PaDEL package [9] were used for modeling. The former contains 4860 chemical substructures that enrich biological activities, and the latter contains 881 binary representations of the chemical structure fragments used by PubChem. 

#### 4.5.2. Feature Selection

To improve the accuracy of the QSAR model and to avoid the overfitting, the feature selection process was performed. In particular, the correlation-based filter method was employed: features with variances that were lower than 0.1 and features demonstrating a high correlation (>0.90) were removed, thus reducing the complexity and computational resources without affecting the model’s performance. 

#### 4.5.3. QSAR Model Construction

**Figure 9:**
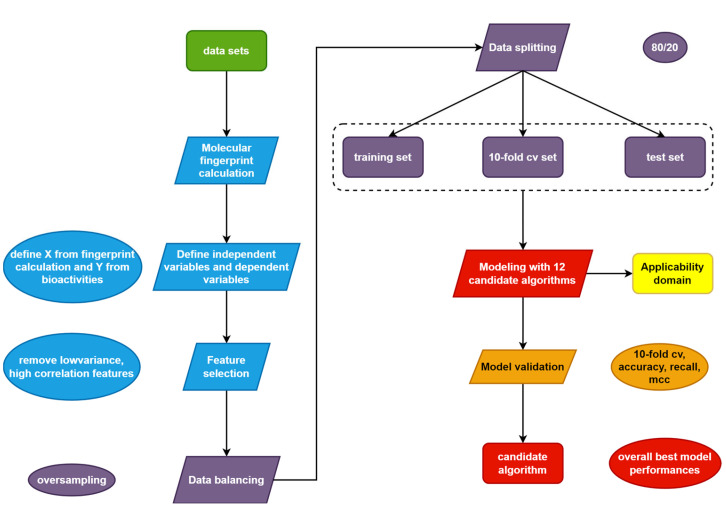
Schematic diagram of the QSAR studies. Different colors represent different stages of the study: green for data compilation, blue for descriptor calculation, purple for data splitting, red for modeling, orange for validation, and yellow for applicability domain determination.

The QSAR models in this study were multiclass classification models that predict 4 bioactivities (potent, active, intermediate, and inactive). Here, the one-vs.-rest strategy was employed for the multi-class classification task. The workflow of the QSAR modeling process is shown in Figure 9. In order to obtain the best model, 12 representative classification algorithms [22,26] have been employed independently for model construction (see Table 7 for a complete list of algorithms). Their performances were evaluated, and the algorithm yielding the best performance will be taken. 

#### 4.5.4. Performance Evaluation and Model Validation

The performance of the QSAR multiclassification models is often evaluated via three parameters: the accuracy, the recall, and the MCC. Let *TP_i_*, *TN_i_*, *FP_i_*, and *FN_i_* denote the true positive, true negative, false positive, and false negative for class *i*, respectively. The accuracy is defined as the number of correct predictions divided by the number of total predictions, i.e., the trace of a confusion matrix divided by the sum of all matrix elements. For the confusion matrix shown in Figure 3a, the sum of the elements in one row gives *TP_i_* + *FN_i_*. Hence, the accuracy reads:

$$\mathrm{Accuracy}\;=\frac{\sum_{i}TP_{i}}{\sum_{i}TP_{i}+FN_{i}}$$

where the index *i* runs through the classes. On the other hand, the micro-averaged recall is defined as [27]:

$$\mathrm{Recall}\;=\underset{i}{\sum }\frac{TP_{i}+FN_{i}}{\sum_{j}TP_{j}+FN_{j}}\cdot \frac{TP_{i}}{TP_{i}+FN_{i}}=\frac{\sum_{i}TP_{i}}{\sum_{j}TP_{j}+FN_{j}}.$$

Again, the indices *i* and *j* run through all classes. Clearly, accuracy and recall are identical in the multiclass cases, and only one needs to be reported. As one extra metric, multiclass MCC is defined as [28]:

$$\mathrm{MCC}=\frac{\left(\sum_{i}TP_{i}\right)\left(\sum_{i}TP_{i}+FN_{i}\right)-\sum_{i}\left(TP_{i}+FN_{i}\right)\left(TP_{i}+FP_{i}\right)}{\sqrt{{\left(\sum_{i}TP_{i}+FP_{i}\right)}^{2}-\sum_{i}{\left(TP_{i}+FP_{i}\right)}^{2}}\sqrt{{\left(\sum_{i}TP_{i}+FN_{i}\right)}^{2}-\sum_{i}{\left(TP_{i}+FN_{i}\right)}^{2}}}.$$

While the values of accuracy and recall range from 0 (worst) to 1 (best), the MCC value ranges from −1 to 1. The extreme values of −1 and 1 represent a perfect misclassification and a perfect classification, respectively. In this study, the balanced steroidal dataset was further split into the training set and the test set according to a ratio of 80:20. Similarly, the same split was performed for most of the nonsteroidal dataset, except for models III and VII, where the splitting was performed with a ratio of 70:30. The size of the training set and the test set from each model is listed in Table 8. Within the training set, a 10-fold cross-validation was performed to guarantee the robustness and reliability of the model. This was done by dividing the training data into 10 groups and using each one for the internal validation, while the other 9 were used to train the model. This process was repeated iteratively until all groups were used for validation.

Out of curiosity, we also performed a binary classification for the best models. By grouping the potent and active data into one group and the intermediate and inactive compounds into the other, we can reduce a 4-by-4 confusion matrix into a 2-by-2 matrix, viz. constructing a binary classification based on the multiclassification results; see Figure S2 in SI. Consequently, we could calculate the random accuracy Q_2,rnd_ [29,30], as well as its deviation from the usual accuracy, i.e., ΔQ_2_, which reflects the impact of data balancing on the model’s performance. If the data are perfectly balanced, its value will be 0.5. Otherwise, this is zero. As this binary classification is out of scope of the present work, the full results are presented in the SI (Table S9), and we mention that our ΔQ_2_ was closer to 0.5, because we always balanced the data before training.

#### 4.5.5. Applicability Domain Determination

The applicability domain of the QSAR models in this study were assessed by means of the bounding box obtained via principal component analysis (PCA). This essentially entails comparing the chemical space of compounds from the training set with those from the test set using the PCA of scores plot. DataWarrior [23] was used for applicability domain determination via PCA. 

### 4.6. Scaffold Analysis

To gain more insights from the structurally diverse 683 nonsteroidal ligands, we applied scaffold analysis to find representative molecular scaffolds. In medicinal chemistry, the molecular scaffold refers to the core structure of a molecule with preferable bioactive properties. Here, the most widely adopted scaffold definition, Murcko-Bemis scaffolding, was employed for the analysis. Murcko and Bemis dissected a molecule into four parts: ring systems, linkers, side chains, and the Murcko framework, which is a union of ring systems and linkers in a molecule [31]. The frequency of occurrences for each scaffold was also ranked for the analysis. DataWarrior [23] was used for scaffold generation and analysis. Three aspects of scaffolds were analyzed.

#### 4.6.1. Murcko Scaffold Visualization

In this study, Murcko scaffolds and cyclic skeleton systems were obtained for non-steroidal androgen receptor antagonists and compared based on pIC_50_ levels so that favorable and unfavorable scaffolds could be identified and further modeled. In addition, the frequency of skeletons and scaffolds was ranked. DataWarrior was used for Murcko scaffold generation and visualization. 

#### 4.6.2. Murcko Scaffold Diversity Analysis

Murcko scaffold diversity was calculated as the proportion of the number of scaffolds to the total number of molecules. 

#### 4.6.3. Scaffold Enrichment Factor Calculation

The scaffold EF is the ratio of the proportion of active molecules with a given scaffold to the proportion of active molecules in the entire dataset [32]. The molecular scaffolds with the highest EF are the most favorable. High-frequency Murcko scaffolds with higher EFs are of particular interest in drug discovery because they can provide more information about the SAR and are enriched with active molecules for the targets of which they have been tested. In this study, the threshold of pIC_50_ = 7.0 (potent, active class threshold) was used for EF calculations. The entire dataset of nonsteroidal molecules had 351 molecules out of 683 with a pIC_50_ ≥ 7.0; therefore, the proportion of active molecules of the entire dataset was 351/683 = 0.51. Furthermore, with the EF value being 1, it is indicative of the same proportion of active molecules in a given scaffold. An EF of 0 means that there are no active molecules in the given scaffold, and an EF of 1.961, with a ratio of 1 to 0.51, means that all molecules in the given scaffold are active. 

### 4.7. Reproducible Research

Reproducibility is defined as the ‘closeness of the agreement between the results of measurements of the same measure and carried out under changed conditions of measurement’. The reproducibility of the experiment, whether in vitro or in silico, is a major concern in science and technology as it is closely related to the extensibility of knowledge and reproducibility of outputs [5]. As this was a computational study, to maintain the reproducibility of the model, all datasets and source codes have been uploaded to the Github repository, and all random seeds were set at 42. All above information can be accessed at https://github.com/GitGears/CYP (accessed on 1 January 2023). Additionally, the prediction results for the training set and the test set of each QSAR model are provided in CSV files in the SI.

## 5. Conclusions

The inhibition of CYP17A1 has become a crucial therapeutic strategy in the treatment of prostate cancer because it reduces androgen levels, which can slow down the growth and spread of prostate cancer cells. This study employed cheminformatics and QSAR modeling for analyzing a set of 962 CYP17A1 inhibitors. In particular, one classification QSAR model was built for steroidal inhibitors, while seven models for major scaffolds with a sufficient number of molecules were built for nonsteroidal inhibitors. All eight QSAR models produced robust performances. The cheminformatic analysis results indicated that most of the 20 representative scaffolds contributed favorably to CYP17A1 inhibition, while only scaffolds 1, 2, 3, 8, and 13 were detrimental to the inhibitory bioactivity of CYP17A1. Furthermore, several functional groups are often employed to modulate the bioactivity of the scaffold, including the hydroxyl group, halogen, fluoride, sulfonamide, ketone, amide, and the methyl group. The effect depends on where the functional group is placed on the scaffold. We hope that the results presented herein will be instrumental for CYP17A1 drug discovery efforts.

## Figures and Tables

**Table 1:** Exploratory data analysis and comparison performed for nonsteroidal and steroidal inhibitors. Six drug-likeness descriptors were employed. They included molecular weight (MW), the octanol-water partition coefficient (LogP), the number of hydrogen bond acceptors (nHA), the number of hydrogen bond donors (nHD), the number of rotatable bonds (nRot), and the topological polar surface area (TPSA).

Results	MW		LogP		nHA	
	Nonsteroidal	Steroidal	Nonsteroidal	Steroidal	Nonsteroidal	Steroidal
***p*-value**	0.17162521		3.01167828 · 10^−16^		2.35402105 · 10^−37^	
**min**	255.05	272.21	1.03	3.13	1	1
**max**	700.33	446.29	6.48	6.18	12	5
**median**	354	363.26	4.02	4.72	5	3
**mean**	380.09	365.59	3.90	4.67	4.69	2.65
**skewness**	1.36	0.56	−0.18	−0.06	1.14	0.55
**kurtosis**	1.49	1.07	−0.13	−0.65	1.54	0.14
**Results**	**nHD**		**nRot**		**TPSA**	
	**Nonsteroidal**	**Steroidal**	**Nonsteroidal**	**Steroidal**	**Nonsteroidal**	**Steroidal**
***p*-value**	0.02433225		1.02741519 · 10^−37^		4.7408051 · 10^−20^	
**min**	0	0	1	0	12.89	12.89
**max**	4	3	13	5	120.27	75.21
**median**	0	1	4	1	49.33	34.89
**mean**	0.65	0.68	4.15	1.49	57.78	38.69
**skewness**	1.53	0.62	0.81	1.92	0.59	0.28
**kurtosis**	1.63	−0.17	0.34	2.51	−0.13	0.52

**Table 2:** Compositions of the top three principal components in terms of the six properties and the accumulated variance.

Property	PC1	PC2	PC3
**MW**	0.383	−0.486	0.270
**LogP**	−0.218	−0.597	0.556
**nHA**	0.535	−0.056	−0.195
**nHD**	0.097	0.578	0.757
**nRot**	0.494	−0.159	0.014
**TPSA**	0.516	0.210	0.081
**Cumulated variance (%)**	54.484	82.646	94.274

**Table 3:** Performance metrics of model I built with the 12 classification algorithms. The abbreviations of the algorithms are defined in the text. The performance is evaluated via the accuracy and MCC. Additionally, the performance is calculated separately for the training set (labeled as Training), the 10-fold cross-validation (labeled as CV), and the test set (labeled as Test). PubChem fingerprint is employed to represent the structures. The best performing algorithm is ET (underlined).

	Accuracy			MCC		
	Training	CV	Test	Training	CV	Test
**DT**	0.933	0.788	0.789	0.911	0.724	0.724
**ET**	0.933	0.818	0.833	0.911	0.766	0.784
**RF**	0.933	0.816	0.844	0.912	0.762	0.798
**GB**	0.933	0.776	0.856	0.911	0.711	0.809
**LGBM**	0.933	0.779	0.844	0.911	0.713	0.796
**XGB**	0.933	0.779	0.833	0.912	0.713	0.783
**SVC**	0.768	0.718	0.667	0.693	0.629	0.559
**MLP**	0.916	0.788	0.844	0.890	0.722	0.798
**LR**	0.760	0.670	0.633	0.681	0.568	0.509
**KNN**	0.763	0.653	0.667	0.690	0.544	0.566
**NB**	0.542	0.514	0.456	0.401	0.365	0.298
**GP**	0.908	0.796	0.811	0.879	0.736	0.751

**Table 4:** Scaffold diversity analysis for nonsteroidal inhibitors. The number of total nonsteroidal compounds, the number of Murcko scaffolds, the number of singleton Murcko scaffolds, and the number of cyclic skeletons are denoted by N, Ns, Nss, and Ncsk, respectively.

	N	Ns	Nss	Ncsk	Ns/N	Nss/N	Ncsk/N	Ncsk/Ns
Complete	683	268	162	150	0.392	0.237	0.220	0.560
pIC_50_ ≥ 7.0	351	165	105	91	0.470	0.299	0.259	0.552
pIC_50_ < 7.0	332	151	101	101	0.455	0.304	0.304	0.627

**Table 5:** List of the effects on functional group substitutions on core fragments, as shown in Figure 4c. Fragments with only one single substitution are not listed but discussed in the text.

Scaffold ID—Core ID	Effect of Substitutions
1-2	R4 position: hydroxy group can increase bioactivities, but nitrogen/sulfur/halogen groups reduce activities.
2-1	Hydroxyl group on R2 or R3 position can enhance bioactivities.
3-1, 3-2, 3-3	R1 position with hydroxyl group yields strong activities; R2 position with fluoride weakens bioactivities.
4-1	R3 position with halogens increases bioactivities; R2 position with sulfonamides decreases bioactivities.
5-1	R1 position with halogens has negative impacts on activities.
7-1	R2 position: sulfonamide is a plus to the activity, but amidine is a minus.
8-1	R1 position must be a bromide to be bioactive.
11-1	R1 position: sulfonyl group contributes positively to bioactivities, while the ketone group contributes negatively.
11-2	R1 position: ketone group contributes positively to activities.
12-1	R1 or R2 positions: hydroxyl and amide groups increase bioactivities, while the carbamate group reduces activities. The length of the sidechain also contributes negatively to activities.
13-1, 13-2, 13-3	Any substitution on the R1 or R2 position will reduce activities.
14-1	R1 or R2 positions: compounds with methyl groups are active.
16-1, 16-2, 17-1, 18-1, 19-1, 19-2, 19-3, 19-5	R1 or R2 positions: compounds with fluoride-containing groups are active.

**Table 6:** Performance metrics for the QSAR classification models of seven groups of scaffolds. Each group contains scaffolds with the same cyclic skeleton. KRC, KR, and PC are the abbreviations for KlekotaRothCount, KlekotaRoth, and PubChem fingerprints, respectively. The accuracy performance is listed for the training set (Training), 10-fold cross-validation (CV), and the test set (Test).

Model	Scaffold	Fingerprint	Algorithm	Accuracy		
				Training	CV	Test
**II**	1,4	KRC	ET	0.978	0.842	0.783
**III**	2,12	KR	RF	0.987	0.771	0.706
**IV**	6,7,11,20	KRC	XGB	0.940	0.741	0.735
**V**	3	KRC	RF	0.977	0.907	0.970
**VI**	5	PC	GP	0.964	0.879	0.905
**VII**	16,17,18,19	KRC	XGB	0.929	0.854	0.888
**VIII**	13	PC	RF	0.960	0.920	0.913

**Table 7:** Machine learning algorithms for modeling.

Algorithm	Abbr	Type	Description
**Decision tree**	DT	Tree model	Tree-structured decision support tool, both for classification and regression models Both classification and regression.
**Extra trees**	ET	Ensemble learning	Extremely randomized trees. Meta-estimator consisting of a multitude of decision trees. Predictions are conducted by averaging the prediction of trees in regression tasks or using majority voting in classification tasks. Unlike random forests that develop each decision tree from a bootstrap sample of the training set, it fits each decision tree upon the entire training dataset. Both classification and regression.
**Random forest**	RF	Ensemble learning	Meta-estimator consisting of a multitude of decision trees, making predictions by averaging the decision tree predictions. Fits each decision tree on a bootstrap sample of the training set. Belongs to the bagging ensemble algorithm. Both classification and regression.
**Gradient boost**	GB	Ensemble learning	Boosting ensemble algorithm, the generalization of AdaBoost. A forward-learning ensemble algorithm that obtains predictive results using gradually improved estimations. Both classification and regression.
**LightGBM**	LGBM	Ensemble learning	Light gradient-boosting machine. A gradient-boosting algorithm based on decision trees to increase the efficiency of the model and reduce memory usage. Characterized by vertical pruning decision trees, high speed, and low memory use. Suitable for large datasets. Both classification and regression.
**Extreme gradient boost**	XGB	Ensemble learning	Extreme gradient boosting. A tree-based ensemble machine learning algorithm that is a scalable, optimized distributed machine learning system for tree boosting. Both classification and regression.
**Multilayer perceptron**	MLP	Artificial neural network	Consists of input and output layers, along with a multitude of hidden layers between. Each node amongst layers is a neuron that utilizes an activation function. Backpropagation tactics are the algorithms for training. Both classification and regression.
**Logistic regression**	LR	Linear model	Modeling the relationship between independent variables and dependent variables by fitting a linear equation to the dataset Classification.
**K-nearest neighbor**	KNN	Non-parametric	A simple algorithm that stores all available cases and predicts the numerical target based on a similarity measure. Both classification and regression.
**Support vector machine**	SVM	Kernel function	Support vector machine constructs a hyperplane in multidimensional space to separate different classes. SVM generates hyperplanes in an iterative manner to minimize an error. Both classification and regression.
**Naive-Bayes**	NB	Naive-bayes	Naive-Bayes classifier is a simple and quick classifier based on probability. Classification.
**Gaussian process**	GP	Non-parametric	Nonparametric Bayesian algorithm that infers a probability distribution over all possible values. Both classification and regression.

**Table 8:** Size of the training and test sets for each QSAR model in this work.

Model	I	II	III	IV	V	VI	VII	VIII
Training	358	89	78	134	129	83	184	275
Test	90	23	34	34	33	21	80	69

## Data Availability

Data can be accessed at https://github.com/GitGears/CYP (accessed on 1 January 2023). Prediction results for the training set and the test set of each QSAR model are provided in CSV files in the SI.

## Supplementary Materials

The following supporting information can be downloaded at: https://www.mdpi.com/article/10.3390/molecules28041679/s1, Figure S1: Complete scatter plot of Murcko scaffold vs pIC_50_ for 683 non-steroidal inhibitors; Figure S2: Binary classification confusion matrices for the eight QSAR models; Table S1: Examples of activity cliffs identified in the nonsteroidal compounds; Table S2 to S8: Performance metrics for model II to model VIII; Table S9: Comparison of the accuracy Q_2_, the random accuracy Q_2,rnd_, and their difference ΔQ_2_ for each QSAR model. Additionally, the QSAR prediction results are available in CSV files, which are named according to the model and the dataset, e.g., file “model1-y-test-pred.csv” contains the prediction results of QSAR model I test set.

## References

[1] Teo M.Y., Rathkopf D.E., Kantoff P. (2019). Treatment of Advanced Prostate Cancer. Annu. Rev. Med..

[2] Gomez L., Kovac J.R., Lamb D.J. (2015). CYP17A1 inhibitors in castration-resistant prostate cancer. Steroids.

[3] Nevedomskaya E., Baumgart S.J., Haendler B. (2018). Recent Advances in Prostate Cancer Treatment and Drug Discovery. Int. J. Mol. Sci..

[4] DeVore N.M., Scott E.E. (2012). Structures of cytochrome P450 17A1 with prostate cancer drugs abiraterone and TOK-001. Nature.

[5] Schaduangrat N., Lampa S., Simeon S., Gleeson M.P., Spjuth O., Nantasenamat C. (2020). Towards reproducible computational drug discovery. J. Cheminformatics.

[6] Fjodorova N., Novich M., Vrachko M., Smirnov V., Kharchevnikova N., Zholdakova Z., Novikov S., Skvortsova N., Filimonov D., Poroikov V. (2008). Directions in QSAR modeling for regulatory uses in OECD member countries, EU and in Russia. J. Environ. Sci. Health Part C Environ. Carcinog. Ecotoxicol. Rev..

[7] Piir G., Kahn I., García-Sosa A.T., Sild S., Ahte P., Maran U. (2018). Best Practices for QSAR Model Reporting: Physical and Chemical Properties, Ecotoxicity, Environmental Fate, Human Health, and Toxicokinetics Endpoints. Environ. Health Perspect..

[8] Tropsha A. (2010). Best Practices for QSAR Model Development, Validation, and Exploitation. Mol. Inform..

[9] Yap C.W. (2011). PaDEL-descriptor: An open source software to calculate molecular descriptors and fingerprints. J. Comput. Chem..

[10] Saad F., Fizazi K., Jinga V., Efstathiou E., Fong P.C., Hart L.L., Jones R., McDermott R., Wirth M., Suzuki K. (2015). Orteronel plus prednisone in patients with chemotherapy-naive metastatic castration-resistant prostate cancer (ELM-PC 4): A double-blind, multicentre, phase 3, randomised, placebo-controlled trial. Lancet Oncol..

[11] Madan R.A., Schmidt K.T., Karzai F., Peer C.J., Cordes L.M., Chau C.H., Steinberg S.M., Owens H., Eisner J., Moore W.R. (2020). Phase 2 Study of Seviteronel (INO-464) in Patients with Metastatic Castration-Resistant Prostate Cancer after Enzalutamide Treatment. Clin. Genitourin. Cancer.

[12] Latysheva A.S., Zolottsev V.A., Pokrovsky V.S., Khan I.I., Misharin A.Y. (2021). Novel Nitrogen Containing Steroid Derivatives for Prostate Cancer Treatment. Curr. Med. Chem..

[13] Mostaghel E.A., Marck B.T., Plymate S.R., Vessella R.L., Balk S., Matsumoto A.M. (2011). Resistance to CYP17A1 inhibition with abiraterone in castration-resistant prostate cancer: Induction of steroidogenesis and androgen receptor splice variants. Clin. Cancer Res. Off. J. Am. Assoc. Cancer Res..

[14] Attard G., Reid A.H.M., Auchus R.J., Hughes B.A., Cassidy A.M., Thompson E., Oommen N.B., Folkerd E., Dowsett M., Arlt W. (2012). Clinical and biochemical consequences of CYP17A1 inhibition with abiraterone given with and without exogenous glucocorticoids in castrate men with advanced prostate cancer. J. Clin. Endocrinol. Metab..

[15] Giacinti S., Bassanelli M., Aschelter A.M., Milano A., Roberto M., Marchetti P. (2014). Resistance to abiraterone in castration-resistant prostate cancer: A review of the literature. Anticancer. Res..

[16] Petrunak E.M., Rogers S.A., Aubé J., Scott E.E. (2017). Structural and Functional Evaluation of Clinically Relevant Inhibitors of Steroidogenic Cytochrome P450 17A1. Drug Metab. Dispos. Biol. Fate Chem..

[17] Al-Masoudi N.A., Ali D.S., Saeed B., Hartmann R.W., Engel M., Rashid S., Saeed A. (2014). New CYP17 hydroxylase inhibitors: Synthesis, biological evaluation, QSAR, and molecular docking study of new pregnenolone analogs. Arch. Der Pharm..

[18] Gumede N.J., Nxumalo W., Bisetty K., Escuder Gilabert L., Medina-Hernandez M.J., Sagrado S. (2020). Prospective computational design and in vitro bio-analytical tests of new chemical entities as potential selective CYP17A1 lyase inhibitors. Bioorganic Chem..

[19] Wróbel T.M., Rogova O., Sharma K., Rojas Velazquez M.N., Pandey A.V., Jørgensen F.S., Arendrup F.S., Andersen K.L., Björkling F. (2022). Synthesis and Structure–Activity Relationships of Novel Non-Steroidal CYP17A1 Inhibitors as Potential Prostate Cancer Agents. Biomolecules.

[20] Simeon S., Anuwongcharoen N., Shoombuatong W., Malik A.A., Prachayasittikul V., Wikberg J.E.S., Nantasenamat C. (2016). Probing the origins of human acetylcholinesterase inhibition via QSAR modeling and molecular docking. PeerJ.

[21] Suvannang N., Preeyanon L., Malik A.A., Schaduangrat N., Shoombuatong W., Worachartcheewan A., Tantimongcolwat T., Nantasenamat C. (2018). Probing the origin of estrogen receptor alpha inhibition via large-scale QSAR study. RSC Adv..

[22] Nantasenamat C., Roy K. (2020). Best Practices for Constructing Reproducible QSAR Models. Ecotoxicological QSARs.

[23] Sander T., Freyss J., Von Korff M., Rufener C. (2015). DataWarrior: An open-source program for chemistry aware data visualization and analysis. J. Chem. Inf. Model..

[24] Guha R., Van Drie J.H. (2008). Structure—Activity landscape index: Identifying and quantifying activity cliffs. J. Chem. Inf. Model..

[25] González-Medina M., Méndez-Lucio O., Medina-Franco J.L. (2017). Activity Landscape Plotter: A Web-Based Application for the Analysis of Structure–Activity Relationships. J. Chem. Inf. Model..

[26] Carracedo-Reboredo P., Liñares-Blanco J., Rodríguez-Fernández N., Cedrón F., Novoa F.J., Carballal A., Maojo V., Pazos A., Fernandez-Lozano C. (2021). A review on machine learning approaches and trends in drug discovery. Comput. Struct. Biotechnol. J..

[27] Sokolova M., Lapalme G. (2009). A systematic analysis of performance measures for classification tasks. Inf. Process. Manag..

[28] Gorodkin J. (2004). Comparing two K-category assignments by a K-category correlation coefficient. Comput. Biol. Chem..

[29] Batista J., Vikić-Topić D., Lučić B. (2016). The Difference between the Accuracy of Real and the Corresponding Random Model is a Useful Parameter for Validation of Two-State Classification Model Quality. Croat. Chem. Acta.

[30] Lucic B., Batista J., Bojović V., Lovric M., Sovic A., Beslo D., Nadramija D., Vikić-Topić D. (2019). Estimation of Random Accuracy and its Use in Validation of Predictive Quality of Classification Models within Predictive Challenges. Croat. Chem. Acta.

[31] Bemis G.W., Murcko M.A. (1996). The properties of known drugs. 1. Molecular frameworks. J. Med. Chem..

[32] Manelfi C., Gemei M., Talarico C., Cerchia C., Fava A., Lunghini F., Beccari A.R. (2021). “Molecular Anatomy”: A new multi-dimensional hierarchical scaffold analysis tool. J. Cheminformatics.

