# Supplementary material for: Exploring the Chemical Space of CYP17A1 Inhibitors Using Cheminformatics and Machine Learning
Source: Molecules. 2023 Feb 9;28(4):1679. doi: 10.3390/molecules28041679 (PMC9966999; doi:10.3390/molecules28041679)
Supplement: Supplementary file 1 [file molecules-28-01679-s001.zip › molecules-2094497-supplementary-1Jan2023.pdf]

Supplementary Information: Exploring the chemical space of CYP17A1 inhibitors using cheminformatics and machine learning

Tianshi Yu[1,2], Tianyang Huang[1], Leiye Yu[3], Chanin Nantasenamat[4], Nuttapat Anuwongcharoen[2], Theeraphon Piacham[5], Ruobing Ren[3,6]* and Ying-Chih Chiang[1]*

[1] Kobilka Institute of Innovative Drug Discovery, School of Medicine, The Chinese University of Hong Kong, Shenzhen, Guangdong, 518172, China
[2] Center of Data Mining and Biomedical informatics, Faculty of Medical Technology, Mahidol University, Bangkok, 10700, Thailand
[3] Shanghai Key Laboratory of Metabolic Remodeling and Health, Institute of Metabolism and Integrative Biology, Fudan University, Shanghai 200438, China
[4] Streamlit Open Source, Snowflake Inc., USA
[5] Department of Clinical Microbiology and Applied Technology, Faculty of Medical Technology, Mahidol University, Bangkok 10700, Thailand
[6] Shanghai Qi Zhi Institute, Shanghai 200030, China
*Correspondence: chiangyc@cuhk.edu.cn; renruobing@fudan.edu.cn.

This file contains two supplementary figures (Fig S1, Fig S2) and nine supplementary tables (Table S1 to Table S9). Details of these data are given in the corresponding captions.

Additionally, the SI contains CSV files, which contain the CHEMBL ID (ID/Name) of the ligands, their true classification (class), and the predicted class (prediction). The files are named according to the model (QSAR model I through VIII) and the data set (training or test), e.g. file "model1-y-test-pred.csv" contains the data from QSAR model I test set.

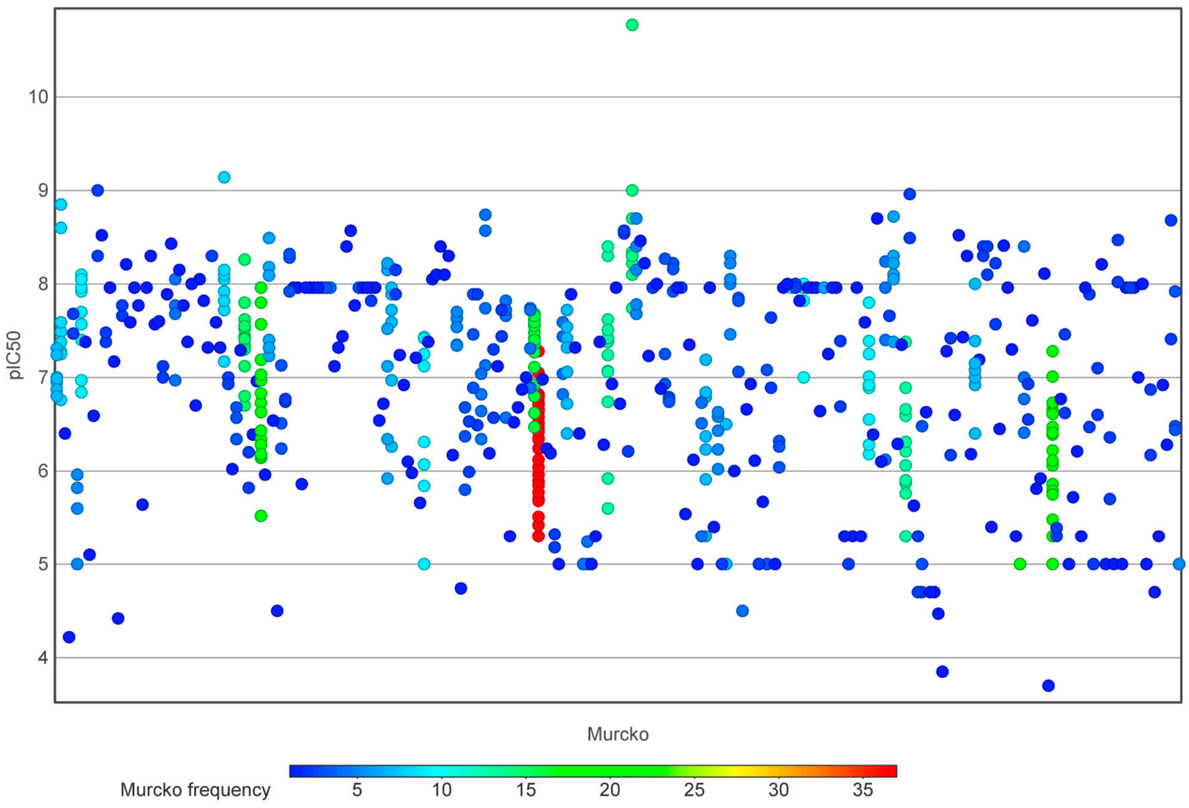

Figure S1. Complete scatter plot of Murcko scaffold vs pIC_{50} for 683 non-steroidal inhibitors.

Table S1. Examples of activity cliffs identified in the nonsteroidal compounds.

Molecule 1	Molecule 2	Similarity	Activity difference	SALI value
CHEMBL4291856<br>pIC_{50}=8.05<br>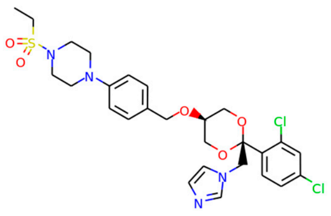	CHEMBL4283221<br>pIC_{50}=5.00<br>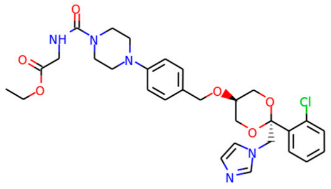	0.870	3.05	23.31
CHEMBL4291856<br>pIC_{50}=8.05<br>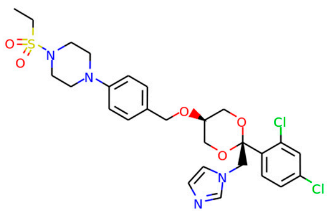	CHEMBL4285744<br>pIC_{50}=5.00<br>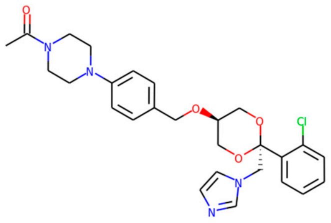	0.899	3.05	30.06
CHEMBL4130227<br>pIC_{50}=10.77<br>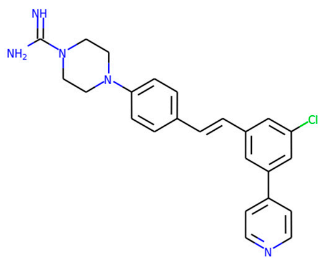	CHEMBL4128197<br>pIC_{50}=7.74<br>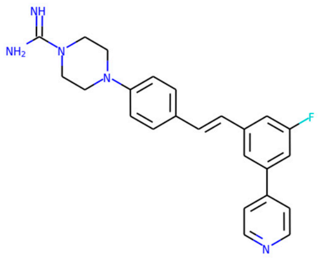	0.91	3.03	32.25

CHEMBL4130227<br>pIC_{50}=10.77<br>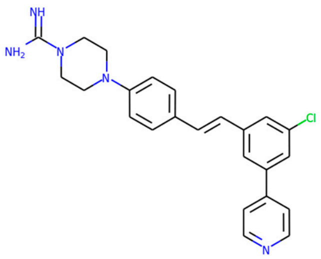	CHEMBL4287119<br>pIC_{50}=6.21<br>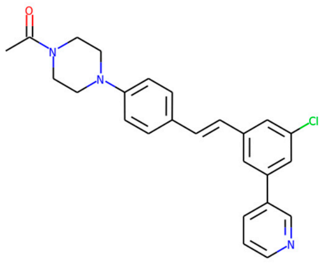	0.867	4.56	34.09
CHEMBL4289930<br>pIC_{50}=8.10<br>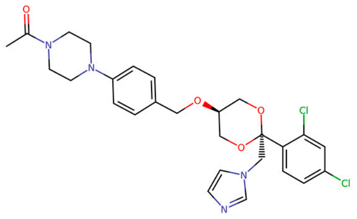	CHEMBL4283221<br>pIC_{50}=5.00<br>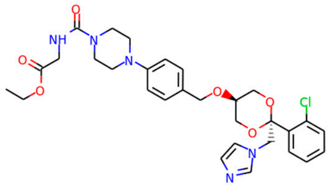	0.932	3.10	45.39
CHEMBL4289930<br>pIC_{50}=8.10<br>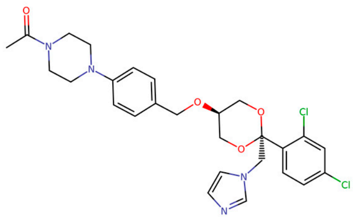	CHEMBL4285744<br>pIC_{50}=5.00<br>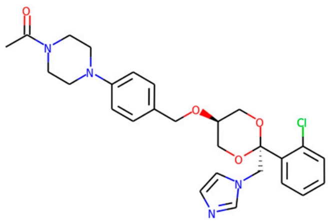	0.975	3.10	122.14

Table S2. Performance metrics for model II, which incorporates molecules containing scaffolds 1 and 4. The KlekotaRothCount fingerprint is used (train:test = 8:2, variance threshold = 0.10, correlation threshold = 0.90, random state = 42). For model II, the best algorithm is ET.

	Accuracy			MCC		
	**Train**	**CV**	**Test**	**Train**	**CV**	**Test**
**DT**	0.978	0.787	0.783	0.971	0.741	0.708
**ET**	<u>0.978</u>	<u>0.842</u>	<u>0.783</u>	<u>0.971</u>	<u>0.802</u>	<u>0.708</u>
**RF**	0.978	0.832	0.783	0.971	0.789	0.712
**GB**	0.978	0.831	0.826	0.971	0.792	0.767
**LGBM**	0.955	0.833	0.826	0.941	0.795	0.767
**XGB**	0.978	0.821	0.826	0.971	0.772	0.767
**SVC**	0.719	0.683	0.609	0.635	0.598	0.472
**MLP**	0.955	0.821	0.783	0.941	0.771	0.712
**LR**	0.899	0.797	0.739	0.867	0.741	0.656
**KNN**	0.753	0.651	0.696	0.672	0.561	0.595
**NB**	0.73	0.619	0.522	0.693	0.536	0.373
**GP**	0.978	0.822	0.739	0.971	0.771	0.656

Table S3. Performance metrics for model III, which incorporates molecules containing scaffolds 2 and 12. The KlekotaRoth fingerprint is used (train:test = 7:3, variance threshold = 0.10, correlation threshold = 0.90, random state = 42). RF performs the best for model III.

	**Accuracy**			**MCC**		
	**Train**	**CV**	**Test**	**Train**	**CV**	**Test**
**DT**	0.987	0.729	0.559	0.983	0.651	0.440
**ET**	0.987	0.757	0.676	0.983	0.693	0.572
**RF**	0.987	0.771	0.706	0.983	0.709	0.606
**GB**	0.987	0.677	0.559	0.893	0.588	0.428
**LGBM**	0.974	0.718	0.647	0.966	0.652	0.562
**XGB**	0.987	0.730	0.647	0.983	0.650	0.522
**SVC**	0.910	0.770	0.618	0.881	0.710	0.522
**MLP**	0.987	0.796	0.676	0.983	0.742	0.556
**LR**	0.923	0.757	0.471	0.897	0.692	0.300
**KNN**	0.769	0.618	0.500	0.690	0.495	0.402
**NB**	0.808	0.714	0.500	0.752	0.634	0.360
**GP**	0.962	0.734	0.676	0.949	0.661	0.583

Table S4. Performance metrics for model IV, which incorporates molecules containing scaffolds 6, 7, 11 and 20. The KlekotaRothCount fingerprint is used (train:test = 8:2, variance threshold = 0.10, correlation threshold = 0.90, random state = 42). XGB performs the best for this model.

	Accuracy			MCC		
	**Train**	**CV**	**Test**	**Train**	**CV**	**Test**
**DT**	0.94	0.696	0.735	0.921	0.606	0.641
**ET**	0.94	0.696	0.647	0.921	0.606	0.539
**RF**	0.94	0.696	0.676	0.92	0.609	0.561
**GB**	0.94	0.688	0.735	0.921	0.597	0.641
**LGBM**	0.933	0.718	0.735	0.911	0.638	0.644
**XGB**	0.94	0.741	0.735	0.921	0.665	0.642
**SVC**	0.701	0.53	0.588	0.61	0.388	0.486
**MLP**	0.91	0.71	0.618	0.881	0.623	0.504
**LR**	0.866	0.642	0.676	0.822	0.543	0.571
**KNN**	0.731	0.575	0.559	0.649	0.454	0.426
**NB**	0.41	0.366	0.382	0.244	0.163	0.245
**GP**	0.933	0.681	0.618	0.911	0.59	0.506

Table S5. Performance metrics for model V, which incorporates molecules containing scaffold 3. The KlekotaRothCount fingerprint is used (train:test = 8:2, variance threshold = 0.10, correlation threshold = 0.90, random state = 42). RF performs the best in model V.

	Accuracy			MCC		
	Train	CV	Test	Train	CV	Test
DT	0.977	0.899	0.97	0.966	0.859	0.956
ET	0.977	0.899	0.97	0.966	0.856	0.956
RF	0.977	0.907	0.97	0.966	0.87	0.956
GB	0.977	0.899	0.97	0.966	0.856	0.956
LGBM	0.961	0.907	0.848	0.942	0.868	0.794
XGB	0.977	0.899	0.97	0.966	0.856	0.956
SVC	0.86	0.783	0.818	0.809	0.693	0.744
MLP	0.938	0.884	0.879	0.909	0.836	0.832
LR	0.86	0.829	0.818	0.795	0.756	0.731
KNN	0.837	0.737	0.697	0.761	0.623	0.558
NB	0.667	0.636	0.727	0.569	0.502	0.67
GP	0.961	0.876	0.97	0.943	0.826	0.956

Table S6. Performance metrics for model VI, which incorporates molecules containing scaffold 5. The PubChem fingerprint is used (train:test = 8:2, variance threshold = 0.10, correlation threshold = 0.90, random state = 42). The best algorithm is GP.

	**Accuracy**			**MCC**		
	**Train**	**CV**	**Test**	**Train**	**CV**	**Test**
**DT**	0.976	0.808	0.762	0.968	0.776	0.692
**ET**	0.976	0.856	0.905	0.968	0.826	0.867
**RF**	0.976	0.867	0.905	0.968	0.84	0.867
**GB**	0.976	0.856	0.905	0.968	0.824	0.867
**LGBM**	0.964	0.808	0.905	0.953	0.777	0.867
**XGB**	0.976	0.867	0.905	0.968	0.841	0.867
**SVC**	0.916	0.856	0.81	0.888	0.82	0.788
**MLP**	0.952	0.881	0.81	0.936	0.853	0.788
**LR**	0.928	0.892	0.81	0.904	0.871	0.788
**KNN**	0.892	0.856	0.857	0.856	0.82	0.832
**NB**	0.855	0.761	0.81	0.81	0.697	0.788
**GP**	0.964	0.879	0.905	0.953	0.855	0.867

Table S7. Performance metrics for model VII, which incorporates molecules containing scaffolds 16, 17, 18 and 19. The KlekotaRothCount fingerprint is used (train:test = 7:3, variance threshold = 0.10, correlation threshold = 0.90, random state = 42). The best algorithm is XGB.

	**Accuracy**			**MCC**		
	**Train**	**CV**	**Test**	**Train**	**CV**	**Test**
**DT**	0.935	0.843	0.85	0.915	0.803	0.809
**ET**	0.935	0.837	0.862	0.915	0.799	0.824
**RF**	0.935	0.826	0.838	0.915	0.785	0.793
**GB**	0.935	0.859	0.888	0.915	0.822	0.856
**LGBM**	0.929	0.848	0.825	0.908	0.812	0.776
**XGB**	<u>0.929</u>	<u>0.854</u>	<u>0.888</u>	<u>0.908</u>	<u>0.819</u>	<u>0.856</u>
**SVC**	0.69	0.665	0.775	0.612	0.584	0.702
**MLP**	0.908	0.811	0.875	0.881	0.757	0.841
**LR**	0.87	0.796	0.888	0.828	0.737	0.851
**KNN**	0.853	0.752	0.788	0.813	0.688	0.733
**NB**	0.679	0.685	0.7	0.625	0.634	0.65
**GP**	0.913	0.837	0.838	0.887	0.8	0.793

Table S8. Performance metrics for model VIII, which incorporates molecules containing scaffold 13. The PubChem fingerprint is used (train:test = 8:2, variance threshold = 0.10, correlation threshold = 0.90, random state = 42). The best algorithm is RF.

	**Accuracy**			**MCC**		
	**Train**	**CV**	**Test**	**Train**	**CV**	**Test**
**DT**	0.96	0.884	0.913	0.947	0.853	0.891
**ET**	0.96	0.92	0.928	0.947	0.899	0.908
**RF**	0.96	0.92	0.913	0.947	0.898	0.891
**GB**	0.96	0.905	0.899	0.947	0.879	0.874
**LGBM**	0.96	0.894	0.899	0.947	0.866	0.874
**XGB**	0.96	0.902	0.899	0.947	0.874	0.874
**SVC**	0.818	0.745	0.754	0.76	0.668	0.671
**MLP**	0.949	0.865	0.884	0.933	0.829	0.858
**LR**	0.836	0.767	0.826	0.783	0.7	0.769
**KNN**	0.858	0.716	0.768	0.813	0.637	0.708
**NB**	0.702	0.698	0.638	0.606	0.606	0.517
**GP**	0.956	0.851	0.899	0.943	0.809	0.874

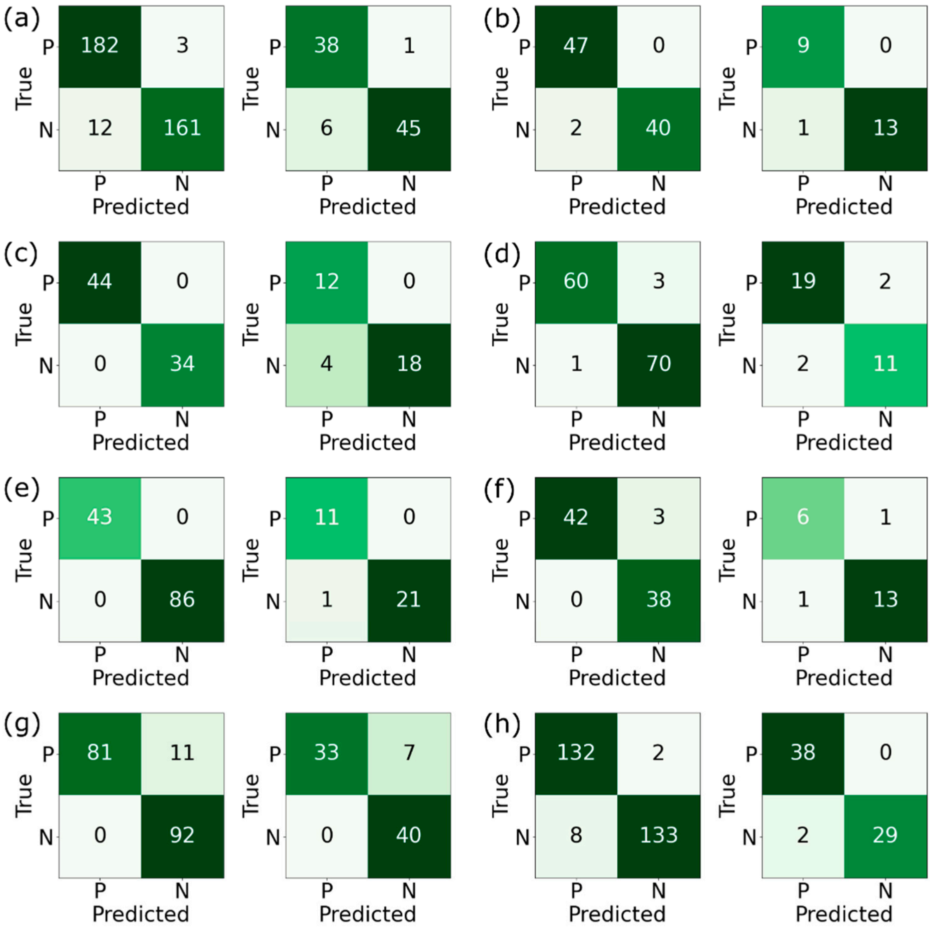

Figure S2. Binary classification confusion matrices for the eight QSAR models, obtained by combining the potent/active elements into group P and the intermediate/inactive elements into group N. Panel (a) to (h) show the confusion matrices for training set (left) and for test set (right) of model I to model VIII, respectively.

Table S9. Comparison of the accuracy Q_2, the random accuracy $Q_{2,rnd}$, and their difference ΔQ_2 for each QSAR model. Calculations were carried out based on binary confusion matrix shown in **Fig S2**. The maximum value of ΔQ_2 is 0.5, which occurs when the numbers of data in each class are well-balanced. In contrast, ΔQ_2=0 occurs when the numbers of data are extremely imbalanced. Most of the ΔQ_2 have a large value, closer to 0.5, indicating that our data are balanced in each class.

	**Training set**			**Test set**		
	**Q_2**	**$Q_{2, rnd}$**	**ΔQ_2**	**Q_2**	**$Q_{2, rnd}$**	**ΔQ_2**
**Model I**	0.958	0.501	0.457	0.922	0.501	0.421
**Model II**	0.978	0.503	0.475	0.957	0.514	0.443
**Model III**	1.000	0.508	0.492	0.882	0.509	0.373
**Model IV**	0.970	0.503	0.467	0.882	0.528	0.354
**Model V**	1.000	0.556	0.444	0.970	0.545	0.425
**Model VI**	0.964	0.501	0.463	0.905	0.556	0.349
**Model VII**	0.940	0.500	0.440	0.913	0.500	0.413
**Model VIII**	0.964	0.500	0.464	0.971	0.508	0.463

Let TP, TN, FP, FN denote true positive, true negative, false positive, false negative. The accuracy for a binary classification reads,

$$Q_2 = \frac{TP + TN}{TP + TN + FP + FN},$$

while the random accuracy reads [1],

$$Q_{2,rnd} = \frac{(TP+FP)(TP+FN)+(TN+FP)(TN+FN)}{(TP+TN+FP+FN)^2}.$$

Their difference, ΔQ_2, is given by,

$$\Delta Q_2 = Q_2 - Q_{2,rnd}.$$

[1] Lucic B, Batista J, Bojović V, Lovric M, Sovic A, Beslo D, et al. Estimation of Random Accuracy and its Use in Validation of Predictive Quality of Classification Models within Predictive Challenges. Croatica Chemica Acta. 2019;92.
